# A cluster analysis of serious adverse event reports after human papillomavirus (HPV) vaccination in Danish girls and young women, September 2009 to August 2017

**DOI:** 10.2807/1560-7917.ES.2019.24.19.1800380

**Published:** 2019-05-09

**Authors:** Daniel Ward, Nicklas Myrthue Thorsen, Morten Frisch, Palle Valentiner-Branth, Kåre Mølbak, Anders Hviid

**Affiliations:** 1 Department of Epidemiology Research, Statens Serum Institut, Copenhagen, Denmark; 2 Department of Clinical Medicine, Center for Sexology Research, Aalborg University, Aalborg, Denmark; 3 Division of Infectious Diseases Preparedness, Statens Serum Institut, Copenhagen, Denmark; 4 Department of Veterinary and Animal Sciences, University of Copenhagen, Denmark

**Keywords:** human papillomavirus, HPV, vaccines, immunisation, diseases, syndromes, conditions, Denmark, surveillance, epidemiology

## Abstract

**Background:**

Suspected adverse events (AE) after human papillomavirus (HPV) vaccines include postural orthostatic tachycardia syndrome (POTS), chronic fatigue syndrome (CFS), complex regional pain syndrome (CRPS) and symptoms including headache and orthostatic intolerance.

**Aim:**

We aimed to identify phenotypes of AEs after HPV vaccination, defined as patterns of AE terms (signs, symptoms, diagnoses), and to evaluate if identified phenotypes reflected previously suspected symptomatology or heightened public concerns over HPV vaccine safety since 1 January 2015.

**Methods:**

We conducted a retrospective observational study using latent class cluster analysis of all serious AE reports (n = 963) reported by females residing in Denmark between September 2009 and August 2017. Resulting clusters were characterised according to AE terms associated with POTS, CFS and CRPS before (September 2009–December 2014) and during (January 2015–August 2017) a time of heightened media activity regarding HPV vaccines.

**Results:**

Four clusters of AE reports were distinguished. The most common symptoms were fatigue, dizziness and headache but their frequency varied among clusters. The majority of reports in one cluster were submitted during a period of heightened media activity, including an anomalous spike in submissions in December 2015; a high proportion of these reports included the symptoms cognitive disorder (78%), abdominal pain (77%), dysuria (60%) and sleep disorder (60%).

**Conclusions:**

Non-specific symptoms including headache, fatigue and dizziness feature prominently in serious AE reports from females in Denmark. Our analysis identified a cluster of reports, likely media stimulated, with a focus on symptoms of CFS and POTS.

## Introduction

In 2006, Gardasil – a quadrivalent human papillomavirus (HPV) vaccine targeting HPV types 6, 11, 16 and 18, was first licenced in the European Union. A bivalent vaccine (Cervarix) targeting HPV types 16 and 18 was subsequently licenced in 2007 and a nonavalent vaccine (Gardasil-9) in 2015. In Denmark, there has been a catch-up programme for HPV vaccination for girls aged 13–15 years since 1 October 2008. From 1 January 2009, HPV vaccination was included as part of the national childhood vaccination programme for girls aged 12 years. HPV catch-up programmes ran from 27 August 2012 to 31 December 2013 for women born 1985–92 and from 1 January 2014 to 31 December 2015 for those born 1993–97 [1,2]. Gardasil was used in programmes until February 2016 when Cervarix was phased in and until November 2017, when Gardasil-9 was introduced replacing Cervarix [3,4].

The World Health Organization (WHO) has recommended implementation of HPV vaccination programmes worldwide [5], however, public concern regarding adverse reactions to the vaccines have threatened their successful introduction in some countries. For example, the Ministry of Health, Labour and Welfare in Japan has withdrawn its recommendation for vaccination due to fears of adverse events (AE) [6]. The uptake of the HPV vaccine in Denmark fell sharply following the broadcasting of a documentary about HPV vaccine AEs, ‘De vaccinerede piger’ (The vaccinated girls) in March 2015. HPV vaccine uptake remained low until September 2016, by which time only 41% of girls born in 2003 (due to receive first dose of the vaccine at 12 years) had received the vaccine [7].

In 2013, a case series reported six patients with onset of postural orthostatic tachycardia syndrome (POTS) 6 days–2 months following HPV vaccination [8]. In 2015, a case series from a Danish syncope clinic described patients investigated for suspected autonomic dysfunction after HPV vaccination [9]; symptoms of headache, orthostatic intolerance, fatigue or nausea were reported frequently but not all patients met a diagnosis of POTS. An additional case series from the Danish syncope clinic, described 35 patients diagnosed with chronic fatigue syndrome (CFS) after HPV vaccination [10]. Co-diagnosis of POTS in patients with CFS (range 13–27%) is recognised [11,12] and is likely due to chronic fatigue being a common presenting feature of POTS. In Japan, a case series of 40 female patients aged 11–17 years old described symptoms including headaches, general fatigue and limb pain after HPV vaccination – chronic regional pain syndrome (CRPS) was diagnosed in 18 patients and POTS in four patients including three with both diagnoses [13]. However, these studies have not been supported by more comprehensive studies, such as a nationwide cohort study from Norway, which found no association between HPV vaccination and increased risk of CFS, and a study from the United Kingdom (UK) utilising AE reports, which also found no association [14,15]. A study of AE reports in the Vaccine Adverse Event Reporting System (VAERS) found approximately one case of POTS per 6.5 million HPV vaccine doses distributed in the United States (US) [16].

In September 2013, the Danish authorities raised the POTS signal to the European Medicines Agency (EMA). In November 2015, the EMA concluded in its report that the current evidence did not support a causal link between HPV vaccination and POTS, CRPS or CFS [17]. The EMA also noted that most of the reports of POTS came from the same Danish syncope clinic [9,10]. The documentary ‘De vaccinerede piger’ in March 2015, the EMA report in November 2015 and attendant media coverage of both stimulated public concern and may have influenced AE reports. A study by Chandler et al. using HPV vaccine AE reports excluded those submitted after 1 January 2015 to reduce the effect of media stimulated reporting and found that the AE terms headache, dizziness and fatigue or syncope tended to cluster together [18].

In Denmark, patients and third parties including healthcare professionals and the Patient Compensation Association can report AEs following drug use or vaccination to the Danish Medicines Agency (DMA) via an online form [19]. The DMA keeps a nationwide database of these reports. Each entry in this database includes information on: (i) the suspected vaccine, (ii) the experienced symptoms, signs and diagnoses coded into AE terms using the Medical Dictionary for Regulatory Activities (MedDRA) at the ‘Preferred Term’ level, (iii) self-reported date of exposure, (iv) self-reported date of onset for each AE term, (v) date of report submission, (vi) the reporter qualification (including physician, nurse, lawyer and consumer or other non-healthcare professional) and, (vii) the identity of the patient using a unique identification number used in all other Danish nationwide registers. The DMA classifies AE reports as serious based on fulfilling one of the following criteria: fatal, life-threatening, hospitalisation, disability/incapacity from employment or education, congenital abnormality, or other medically significant events including cases of suspected POTS or two or more syncopal episodes due to their relevance to a putative safety signal.

This study used reports of serious AE following HPV vaccination submitted to the DMA from females aged 5 years and older residing in Denmark. The aim of this study was to identify and describe adverse reaction phenotypes, defined as patterns of AE terms reported to the DMA in serious AE reports following HPV vaccination using latent class cluster analysis. We aimed to evaluate if the previously characterised symptomatology figured prominently in the identified clusters and whether the identified clusters related to heightened public concerns over HPV vaccine safety since 1 January 2015.

## Methods

### A retrospective observational study of adverse event reports

In August 2017, Statens Serum Institut obtained a dataset comprising all serious AE reports after HPV vaccination submitted to the DMA between 12 September 2009 and 17 July 2017 (the first and last reported AE, respectively). A total of 979 unique serious AE reports following HPV vaccination were reported by 976 individuals. The collection of all AE terms reported for one individual relating to one vaccination event constituted one report (see Supplement S1). AE terms consisted primarily of symptoms rather than specific diagnoses. The dataset did not include the reason for each AE report’s classification as serious, but previous studies on the safety profile of HPV vaccines in Denmark found that 17% of reports were from patients whose normal daily activities were impaired following HPV vaccination [20,21].

These reports comprised 28,465 AEs (1,164 unique AE terms). We removed duplicates where multiple persons with different reporter qualifications (e.g. physician and lawyer) reported the same patient and AE terms. We also removed reports from individuals younger than 5 years of age (who were children of women vaccinated in pregnancy) and reports from males. The final resulting dataset consisted of 963 reports from 960 females reporting 15,949 AEs (1,144 unique AE terms). The reports included 864 AE reports for Gardasil, two AE reports for Cervarix and 97 AE reports where the type of HPV vaccine was unspecified.

Of 963 reports, the date of vaccination was incomplete in 65 (7%), 24 only contained month and year, 17 only contained year and 24 had completely missing dates. Danish civil registration numbers i.e. unique personal identification numbers for Danish residents, were used in individual AE reports and are also used in all Danish administrative databases [22]. It was possible therefore, to link the submitted AE reports via this identification number with the Danish Vaccination Registry [23] to obtain a more accurate date of HPV vaccination recorded at the time of vaccination that is not limited by recall error.

Of 15,949 reported AEs, the date of AE onset was incomplete for 11,968 AE terms (75%), 3,144 contained year and month of onset, 2,948 only contained the year and 5,876 had no date at all. We used the date information to estimate time since HPV vaccination to AE term onset and to AE report submission date. We performed date imputation, as performing calculations with only complete AE term onset dates would underestimate time from vaccination to AE term onset and/or time from AE term onset to report submission (Supplement S1). Completely missing AE term onset dates were excluded from date calculations, but these reports were still used for cluster analysis as date information was not required.

### Cluster analysis

We used latent class cluster analysis to estimate clusters (‘latent classes’) of AE reports. Latent class cluster analysis differs from classical clustering methods by being a model-based approach, with the assumption that the observed data has been generated by a mixture of probability distributions [24]. For a pre-specified number of clusters our model assumes that the reports are independent, that each report is assigned to a cluster with some probability and that the AE terms are Bernoulli distributed and are conditionally independent within the clusters. The model parameters estimated are the probability of a report belonging to a certain cluster and the conditional probabilities of each AE term given the cluster using maximum likelihood methods (applying the expectation maximization algorithm combined with the Newton-Raphson method [25]).

A requirement of the model is that the number of clusters and AE terms included in the model must be balanced against the number of reports in the dataset; using *m* clusters and *k* AE terms requires at least m*k + m-1 reports. In our dataset, many AE terms were only present in a few reports, with 857 (75%) of unique AE terms being present in five or fewer reports.

We selected the optimal number of clusters based on the Bayesian information criteria (BIC) [26], and used 200 iterations (each with a random initial value for the expectation maximization algorithm) for each combination of clusters and AE terms to reduce the risk of choosing a local solution to the log-likelihood. We initially performed an analysis with the 48 most frequent symptoms in our dataset, fitting from 1 to 20 clusters. After finding the optimal number of clusters for the 48 most frequent AE terms, we steadily increased the amount of AE terms included in the analysis. The purpose of this was to use as much of the dataset as possible and to evaluate the stability of the cluster analysis if we omitted less frequent AE terms. The final model included four clusters and 192 AE terms.

All cluster analyses were conducted in the programming language R (R Foundation for Statistical computing, Vienna, Austria) using the poLCA package for the cluster analysis [27,28].

### Cluster characterisation

To evaluate the similarity of the resulting clusters with POTS, CRPS and CFS, we selected associated terms from current diagnostic guidelines specific to each syndrome (respectively, the ‘consensus statement’ on POTS [29], the ‘Budapest’ criteria for CRPS [30] and the Institute of Medicine (IOM) CFS diagnostic criteria [31]). IOM has proposed the use of the term ‘systemic exertional intolerance syndrome’ to replace CFS, but as our data was coded using the term ‘chronic fatigue syndrome’ we used this terminology.

The selected POTS terms were ‘postural orthostatic tachycardia syndrome’, ‘orthostatic intolerance’, ‘tachycardia’, ‘palpitations’ and ‘syncope’. The selected CRPS terms were ‘complex regional pain syndrome’, ‘hyperaesthesia’, ‘allodynia’, ‘arthralgia’, ‘muscular weakness’ and ‘hyperhidrosis’. The selected CFS terms were ‘chronic fatigue syndrome’, ‘fatigue’, ‘malaise’, ‘sleep disorder’ and ‘impaired work ability’. The frequency of these terms was compared with the total number of AE terms per report to identify clusters which represented phenotypes similar to those syndromes, independent of diagnosis. We also compared the frequency of the combination of the terms headache, dizziness and either fatigue or syncope (previously suggested to be representative of an autonomic dysfunction syndrome [18]) in reports to investigate whether this phenotype was represented in our clusters.

## Results

### Ages of females reporting serious AEs after HPV vaccination

The median age at HPV vaccination of female patients reporting serious AEs to the DMA was 15.1 years (Table 1). The age at vaccination was bimodally distributed, with a large peak at 12 years reflecting those vaccinated in the childhood programme and a smaller peak at 21 years reflecting those vaccinated as part of the catch-up programmes and by private purchase. The median age at report submission was 21.8 years, which is 6.7 years after the median age at vaccination. However, this apparent large time lag is to some extent caused by the bimodal distribution of both features. Comparing the period before 1 January 2015 against the period after, we observed a decrease in median age at vaccination from 21.1 to 14.5 years, reflecting the increasing number of adolescent girls vaccinated through the national childhood programme, compared with fewer adult women being vaccinated through the catch-up programme, which ended in 2015.

**Table 1:** Characteristics of reports of serious adverse events in females following human papillomavirus vaccination, stratified by age at vaccination, Denmark, 12 September 2009–17 July 2017 (n = 963)

Characteristics	Total	12 September 2009–31 December 2014	1 January 2015–17 July 2017
**Number of serious AE reports**			
All reports	963	305	658
11–17 years	525	130	395
≥ 18 years	403	165	238
Unknown age	35	10	25
**Median age at vaccination in years (IQR)**			
All reports	15.1 (12.5–24.4)	21.1 (12.8–26.4)	14.5 (12.4–23.2)
11–17 years	12.6 (12.1–14.1)	12.6 (12.2–14.0)	12.6 (12.1–14.1)
≥ 18 years	25.4 (22.2–31.5)	25.9 (22.5–31.5)	24.9 (22.0–31.6)
**Median age at report submission in years (IQR)**			
All reports	21.8 (17.1–27.1)	22.8 (16.3–27.9)	21.4 (17.6–26.7)
11–17 years (at vaccination)	17.7 (15.4–20.2)	15.5 (13.8–17.9)	18.4 (16.1–20.8)
≥ 18 years (at vaccination)	27.9 (25.3–33.5)	27.2 (24.2–32.3)	28.3 (26.1–34.8)
**Median time from vaccination to AE term onset in days (IQR)**			
All reports	191 (36–508)	85 (12–297)	223 (58–615)
11–17 years	263 (56–714)	203 (44–479)	292 (67–812)
≥ 18 years	135 (22–333)	63 (5–212)	183 (55–420)
**Median time from AE term onset to report submission in days (IQR)**			
All reports	785 (321–1,471)	306 (113–728)	973 (601–1,774)
11–17 years	1,131 (472–1,873)	674 (201–1,530)	1,265 (630–2,043)
≥ 18 years	601 (207–951)	216 (73–436)	844 (579–1,140)
**Median time from vaccination to report submission in days (IQR)**			
All reports	1,231 (713–2,162)	477 (207–1,142)	1,562 (1,018–2,364)
11–17 years	1,798 (1,052–2,379)	1,059 (350–1,670)	2,162 (1,336–2,473)
≥ 18 years	897 (399–1,212)	352 (174–594)	1,082 (899–1,399)
**Median number of AE terms per report (IQR)**			
All reports	13 (6–22)	9 (4–23)	14 (7–22)
11–17 years	12 (5–22)	10 (3–23)	13 (6–21)
≥ 18 years	14 (6–23)	9 (4–23)	15 (9–24)

AE: adverse event; IQR: interquartile range.

Ages used to stratify are the ages at the time of vaccination.

### Temporal features of reports

**Figure 1:**
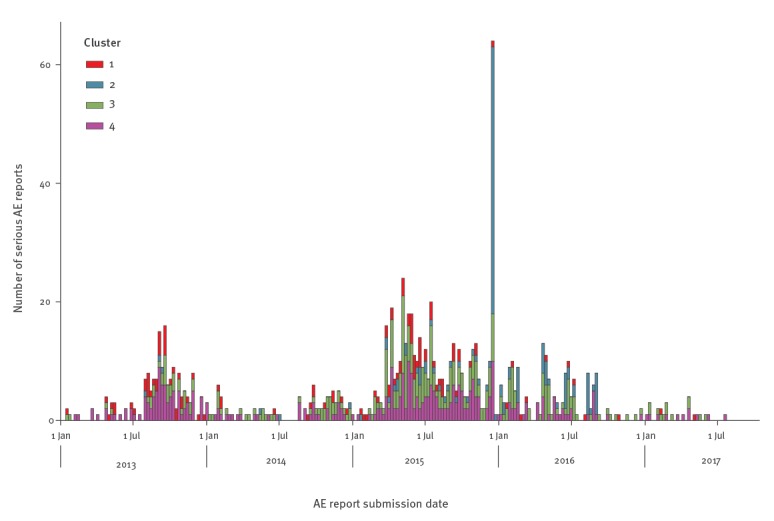
Submission dates of reports of serious adverse events to human papillomavirus vaccination in females, Denmark, January 2013–July 2017 (n = 919)

Median time from vaccination to AE term onset was 191 days and median time from vaccination to report submission was 1,231 days. For reports submitted from 12 September 2009 to 31 December 2014 the median time from vaccination to AE term onset increased from 85 days to 223 days. In total, 305 reports were submitted of which, 139 were between July and December 2013. For reports submitted after 1 January 2015, the median time from vaccination to report submission increased from 477 days to 1,562 days (Table 1). The total number of reports filed was 658, with an anomalous spike comprising 64 reports during a single week in December 2015 (Figure 1).

AE: adverse event.

The height of each bar corresponds to the number of reports, the width of each bar equals 1 week and the colours represent the reports’ cluster. Reports before 1 January 2013 are excluded from the graph as there were only 44 observations.

### Cluster analysis results and characterisation

The cluster analysis identified four clusters of AE reports. All clusters had fatigue, dizziness and headache as the most common AE terms, while three clusters (1, 2, 3) had these terms in the majority (> 50%) of reports (Table 2).

**Table 2:** Ten most common adverse event (AE) terms within clusters of reports of serious AE following human papillomavirus vaccination in females, Denmark, 12 September 2009–17 July 2017 (n = 963)

Rank	Cluster 1 (120 reports)			Cluster 2 (130 reports)			Cluster 3 (329 reports)			Cluster 4 (384 reports)		
	AE term	n	%	AE term	n	%	AE term	n	%	AE term	n	%
1	Fatigue	114	95	Fatigue	120	92	Fatigue	294	89	Headache	142	37
2	Dizziness	113	94	Dizziness	118	91	Dizziness	248	75	Fatigue	126	33
3	Headache	107	89	Headache	111	85	Headache	242	74	Dizziness	104	27
4	Nausea	101	84	Cognitive disorder	102	78	Nausea	186	57	Syncope	71	18
5	Arthralgia	100	83	Abdominal pain	100	77	Arthralgia	149	45	Nausea	53	14
6	Disturbance in attention	99	83	Nausea	98	75	Disturbance in attention	143	43	Arthralgia	47	12
7	Memory impairment	88	73	Muscular weakness	94	72	Abdominal pain	118	36	Disturbance in attention	35	9
8	Muscular weakness	80	67	Palpitations	92	71	Syncope	110	33	Sensory disturbance (tied with 9)	34	9
9	Myalgia	75	63	Dysuria (tied with 10)	78	60	Muscular weakness	101	31	Abdominal pain (tied with 8)	34	9
10	Palpitations	73	61	Sleep disorder (tied with 9)	78	60	Myalgia/memory impairment^a^	100	30	Paraesthesia/ pain/migraine^a^	26	7

AE: adverse event.

^a^ There was a tie between the listed adverse event terms.

Cluster 1 had 120 reports, with high proportions of fatigue (95%), dizziness (94%) and headache (89%) (Table 2). It had a median of 42 AE terms per report, which was the greatest of the four clusters and also had the greatest proportion of reports containing the AE terms ‘POTS’ (30%), and ‘CFS’ (4%) and ‘CRPS’ (1%) (Table 3). Reports contained a median of one POTS-associated AE term, two CRPS-associated AE terms and two CFS-associated AE terms (Table 3). Of 120 reports, the combination of headache, dizziness and syncope or fatigue featured in 82% of reports. Cluster 1 also had the oldest median age at vaccination (19.8 years; IQR: 12.8–26.0) (Table 4).

**Table 3:** Features of POTS, CFS and CRPS in clusters formed from serious adverse event reports following human papillomavirus vaccination in females, Denmark, 12 September 2009–17 July 2017 (n = 963)

Features	Cluster 1 (120 reports)		Cluster 2 (130 reports)		Cluster 3 (329 reports)		Cluster 4 (384 reports)	
	n (IQR)	%	n (IQR)	%	n (IQR)	%	n (IQR)	%
AE terms per report, median	42 (37–53)	NA	20 (17–25)	NA	16 (12–21)	NA	5 (3–7)	NA
Reports containing POTS	36	30	34	26	55	17	15	4
Reports containing CRPS	1	1	0	0	4	1	1	0
Reports containing CFS	5	4	0	0	4	1	2	1
POTS-associated terms per report, median	1 (1–2)	NA	1 (1–2)	NA	1 (0–1)	NA	0 (0–1)	NA
CRPS-associated terms per report, median	2 (1–3)	NA	1 (1–2)	NA	1 (0–1)	NA	0 (0–0)	NA
CFS-associated terms per report, median	2 (1–2)	NA	2 (1–3)	NA	1 (1–2)	NA	0 (0–1)	NA
Reports containing headache, dizziness and either syncope or fatigue	98	82	99	76	181	55	39	10

AE: adverse event; CFS: chronic fatigue syndrome; CRPS: chronic regional pain syndrome; IQR: interquartile range; NA: not applicable; POTS: postural orthostatic tachycardia syndrome.

**Table 4:** Temporal features of clusters of reports of serious adverse events following human papillomavirus vaccination in females, Denmark, 12 September 2009–17 July 2017 (n = 963)

Temporal features	Cluster 1 (120 reports)		Cluster 2 (130 reports)		Cluster 3 (329 reports)		Cluster 4 (384 reports)	
	n (IQR)	%	n (IQR)	%	n (IQR)	%	n (IQR)	%
Reports 12 September 2009–31 December 2014	53	44	6	5	82	25	164	43
Reports 1 January 2015–17 July 2017	67	56	124	95	247	75	220	57
Median age at vaccination (years)	19.8 (12.8–26.0)	NA	16.3 (12.6–23.7)	NA	15.3 (12.5–24.1)	NA	14.2 (12.4–23.9)	NA
Median age at report submission (years)	24.1 (18.5–28.8)	NA	23.5 (18.7–28.0)	NA	21.9 (17.7–27.2)	NA	20.2 (16.3–26.6)	NA
Median time from vaccination to AE onset, (days)	187 (27–444)	NA	172 (27–417)	NA	203 (43–610)	NA	194 (48–432)	NA
Median time from AE term onset to report submission (days)	713 (264–1,516)	NA	1,032 (616–1,959)	NA	856 (390–1,463)	NA	670 (191–1,296)	NA
Median time from vaccination to report submission (days)	1,063 (627–2,079)	NA	1,553 (1,156–2,473)	NA	1,322 (873–2,238)	NA	1,022 (380–1,798)	NA

AE: adverse events; IQR: interquartile range; NA: not applicable.

Cluster 2 had 130 reports, also with high proportions of fatigue (92%), dizziness (91%) and headache (85%), but differed from the other clusters by high proportions of cognitive disorder (78%), abdominal pain (77%), dysuria (60%) and sleep disorder (60%) (Table 2). It also had a high proportion of AE reports containing POTS (26%) (Table 3). Although cluster 2 had no reports with the AE term ‘CFS’, it had a median of two other AE terms considered CFS-associated, which was high relative to the other clusters. Of 130 reports, the combination of headache, dizziness and syncope or fatigue featured in 76% of reports. This cluster had the shortest median period from vaccination to AE term onset at 172 days, but the longest median period from AE term onset to report submission at 1,032 days (Table 4). The majority of reports in this cluster (95%) were submitted after 1 January 2015.

Cluster 3 had 329 reports, with a high proportion of reports with fatigue (89%), dizziness (75%), headache (74%) and nausea (57%), but other terms were reported relatively inconsistently with less than 50% each (Table 2). It had a median number of 16 AE terms per report and the combination of headache, dizziness and syncope or fatigue featured in 55% of reports (Table 3). Although there were 55 reports with POTS, four with CRPS and four with CFS (one report featured both POTS and CFS), these only accounted for 19% of reports.

Cluster 4 had 384 reports, with headache (37%), fatigue (33%) and dizziness (27%) as the most common AE terms although these proportions were low compared to other clusters (Table 2). Sensory disturbance and paraesthesia ranked higher in this cluster compared with other clusters, but were still relatively rare at 9% and 7%, respectively. It had a median of five AE terms per report, and only 5% of reports contained POTS, CRPS or CFS, while 10% had the combination headache, dizziness and syncope or fatigue (Table 3).

**Figure 2:**
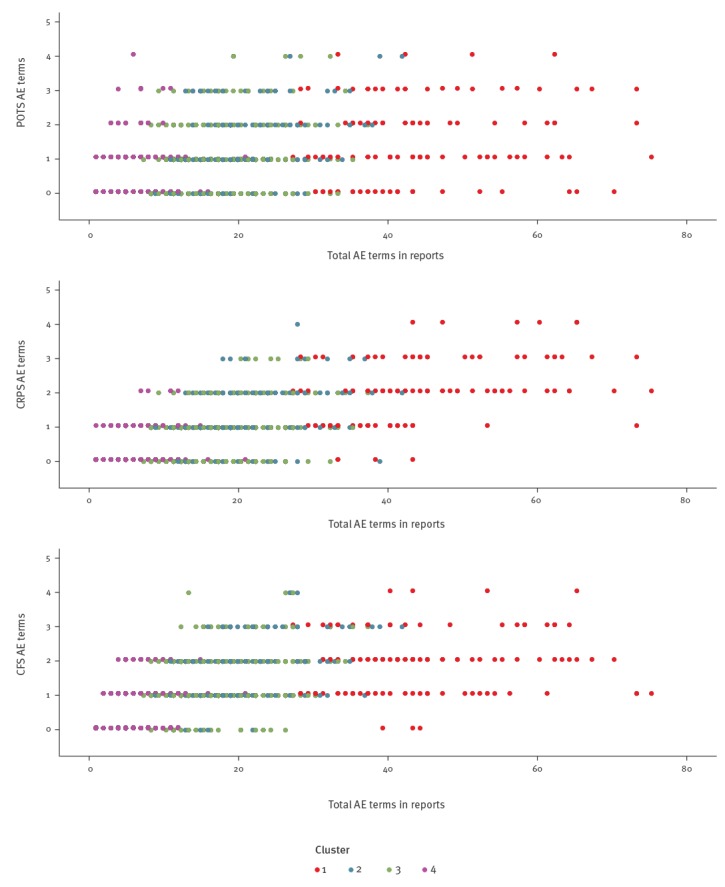
Number of AE terms associated with POTS, CRPS and CFS relative to total number of AE terms in reports of serious AE following HPV vaccination in females, Denmark, 12 September 2009–17 July 2017 (n = 963)

AE: Adverse event; CFS: chronic fatigue syndrome; CRPS: complex regional pain syndrome; HPV: human papillomavirus; POTS: postural orthostatic tachycardia syndrome.

The submission dates of AE reports for clusters 1, 3 and 4 tended to follow a similar temporal pattern, but reporting of AE in cluster 2 was more prominent after 1 January 2015 and was primarily responsible for the anomalous spike in December 2015 with 45 reports (35% of all cluster 2 reports) (Figure 1). Comparing the number of AE terms per report to number of POTS, CFS and CRPS associated terms did not distinguish any close relationship between clusters and selected terms. They visually demonstrated that one of the main features separating the four clusters was the number of AE terms per report, except a close overlap of cluster 2 and 3 (Figure 2).

Each dot represents one or more report and the colours represent the reports’ cluster. Four observations with > 80 adverse event terms per report were excluded to better visualise the clusters.

## Discussion

In our study, fatigue, headache and dizziness were the most common AE terms identified in all four clusters of AE reports, particularly in clusters 1–3; this combination was found in 417 (43%) serious AE reports in our study. A study by Chandler et al. found a similar combination of symptoms i.e. headache, dizziness and fatigue or syncope [18] and a study monitoring quadrivalent HPV vaccines in the VAERS also found that fatigue, headache and dizziness were among the most common AE terms reported from females in the US [32].

Temporal analysis revealed a clear pattern in report submission dates with peaks in 2013 and 2015; both periods coincided with heightened media activity regarding HPV vaccine safety in Denmark, which may have increased reporting of AE. As well as the 2015 documentary about HPV vaccine AE, there were two series of Danish newspaper articles with negative views on HPV vaccination in 2013, which in a study by Suppli et al. were correlated with the decline in HPV vaccine uptake at that time [33]. We also found that cluster 2 consisted mainly of reports submitted after 1 January 2015 and characterised a prominent temporal anomaly, with a relatively large number of reports submitted during a single week in December 2015.

Changes in diagnostic criteria may also be a contributory explanation for the majority of reports in cluster 2 being submitted since 2015. In 2015, IOM revised the diagnostic criteria for CFS, describing ‘cognitive impairment’ and ‘sleep disorder’ as core symptoms, with genitourinary and gastrointestinal symptoms (such as dysuria and abdominal pain) as secondary symptoms [31]. We note that in addition to fatigue, all these symptoms are common in cluster 2.

In order to identify phenotypes of AE reports without explicit diagnoses, we used a selection of AE terms closely associated with POTS, CRPS and CFS, based on current diagnostic guidelines. We selected AE terms specific to each syndrome, however, the inherent non-specific nature of these syndromes weakens the discriminatory ability of the selected AE terms for these case definitions. For example, fatigue, while essential to CFS, features in many illnesses which would require exclusion before a diagnosis of CFS can be made.

Caution is warranted in the interpretation of spontaneous AE reports, especially following the introduction of a new vaccine into a population. From public health surveys we know that a non-trivial proportion of adolescent girls will experience symptoms shortly after vaccination due to chance alone [34]. In addition, the majority of reported symptoms e.g. headaches, dizziness etc. are not uncommon in adolescents. In a large health survey of Danish schoolchildren, 30% of all girls aged 13 years experienced headaches, 20% reported dizziness, 46% sleep disorder and 20% stomach aches on a weekly basis [35]. The results from that survey, suggest that the symptomatology of these girls is similar to that found in our AE data following HPV vaccination. Observational studies of HPV AE have not substantiated any association with CFS, while studies in Norway and Finland have documented an increase in the occurrence of CFS which occurred independently of HPV vaccination [14,15,36].

This study had several limitations. First, we used data from spontaneous reports specifically when determining the exact date of AE term onset, which was often missing. This, together with the delay between vaccination date to the date of AE report submission in the majority of reports, prevents the identification of a relevant latency period between vaccination and AE onset and weakens any potential safety signal that might be implied. However, the incomplete date information did not impact the validity of the results as date information was not used in the cluster analysis. AE report narratives and medical records were not available for review; therefore, we were unable to validate AE reports independently.

Second, the cluster analysis method used restricted the number of AE terms and clusters allowed. While our results remained robust when excluding more AE terms than dictated by the total number of reports available for analysis, it is possible that we did not identify small clusters characterised by rare AE terms.

Finally, we were unable to analyse by vaccine type, this was due to the large number of reports with unspecified HPV vaccine type and the requirement of the clustering method. However, the majority of vaccine types reported were Gardasil and as the Danish vaccination programme used Gardasil exclusively until February 2016, it can be assumed that the majority of unspecified vaccine types were Gardasil.

## Conclusion

In our study, cluster analysis identified a group of AE reports (cluster 2) likely resulting from stimulated reporting due to increased focus on symptoms of CFS and POTS in the clinical setting and in the media. Headache, dizziness and fatigue were prominent features of all four clusters identified from AE reports following HPV vaccination of girls and young women. These are mainly non-specific symptoms and are common occurrences in the target population, so the causation of these symptoms by the HPV vaccine cannot be confirmed by this analysis. We found little support for clusters representing CFS or CRPS, nor any novel autonomic dysfunction syndrome; POTS featured explicitly in many reports in clusters 1–3 (17–30%), but to an extent compatible with the fact that the DMA has specifically designated reports of POTS as serious AEs.

This study indicates the utility of cluster analysis in identifying groups of reports that may relate to stimulated reporting and specific diagnostic practices. Cluster analysis is a relatively new method in epidemiology and further validation and advancement of this technique is warranted. Spontaneous reporting systems and the interpretation of safety signals can be improved with greater validation of details of reports such as vaccine type and onset of AEs, and further understanding of the relationship between reporting patterns and media activity.

## Supplementary Data

23000 Supplement S1
