# Supplementary material for: A cluster analysis of serious adverse event reports after human papillomavirus (HPV) vaccination in Danish girls and young women, September 2009 to August 2017
Source: Euro Surveill. 2019 May 9;24(19):1800380. doi: 10.2807/1560-7917.ES.2019.24.19.1800380 (PMC6518966; doi:10.2807/1560-7917.ES.2019.24.19.1800380)
Supplement: Supplement S1 [file 1800380_WARD_SupplementS1.docx]

**Supplement S1. Explanation of the dataset and handling of missing data**

This supplementary material is hosted by Eurosurveillance as supporting information alongside the article “A cluster analysis of serious adverse event reports after human papillomavirus (HPV) vaccination in Danish girls and young women” on behalf of the authors who remain responsible for the accuracy and appropriateness of the content. The same standards for ethics, copyright, attributions and permissions as for the article apply. Eurosurveillance is not responsible for the maintenance of any links or email addresses provided therein.

**Adverse event report dataset structure**

The initial dataset of reports of suspected adverse events came in a format where each adverse event (AE) term corresponded to one row of data, with each report possibly spanning multiple rows when it contained multiple AE terms. **See example in Table S1 (not using actual data and not all variable columns are included).**

**Table S1: Adverse event report dataset example.**

| **ID of person experiencing suspected AE** | **Date of report submission** | **AE term** | **Vaccination date** | **AE term onset date** |
| --- | --- | --- | --- | --- |
| 1 | 12/05/2012 | HEADACHE | 15/07/2011 | 12/09/2011 |
| 2 | 08/11/2015 | ARTHRALGIA | 05/01/2012 | 2013 |
| 2 | 08/11/2015 | HEADACHE | 05/01/2012 | NA |
| 2 | 08/11/2015 | SYNCOPE | 05/01/2012 | 01/2014 |
| 3 | 21/02/2013 | FATIGUE | 08/2012 | NA |
| 3 | 21/02/2013 | DIZZINESS | 08/2012 | 03/11/2012 |

Note that date of report submission and vaccination date were fixed for each report. For the cluster analysis, this was transformed into a data format coding presence or absence of symptoms as 2 and 1 respectively (1 and 0 are more commonly used, but the poLCA package requires that variables to be clustered on use strictly positive integers). In Table S2 we see how adverse event report data from Table S1 was formatted for cluster analysis.

**Table S2: Cluster analysis dataset example.**

| **ID of person experiencing suspected AE** | **HEADACHE** | **ARTHRALGIA** | **SYNCOPE** | **FATIGUE** | **DIZZINESS** |
| --- | --- | --- | --- | --- | --- |
| 1 | 2 | 1 | 1 | 1 | 1 |
| 2 | 2 | 2 | 2 | 1 | 1 |
| 3 | 1 | 1 | 1 | 2 | 2 |

The ID was retained to subsequently match the assigned cluster back to the original dataset correctly, but was not used in the cluster analysis. The cluster analysis was then performed using the poLCA function in R from the poLCA package. The model formula used was cbind (HEADACHE, FATIGUE,…) ~ 1 using the n most common symptoms for various values of n (192 in the final model reported on in the article).

**Imputation of AE term onset date information**

Date information was partially incomplete for 38% of all AE term onset dates and completely missing for 37%. We performed date imputation on partially incomplete dates for the description of temporal patterns. Conducting temporal analysis only on observations with complete date information would introduce bias. In our data, completeness of dates typically corresponds to shorter time between vaccination and AE report submission (Table S3). Consequently, analyses of temporal patterns with only complete AE term onset dates would underestimate time from vaccination to AE term onset, and/or time from AE term onset to report. As different AE terms within a report could have different dates of onset, and a substantial proportion of AE term onset dates were partially missing, we imputed incomplete dates using the conditional distribution of onset times without contradicting the partial information provided. When the conditional dataset had no observations, we performed imputation using a uniform distribution of the possible onset times given the partial information. In cases where the given date of AE term onset was prior to vaccination, we used a 7-day grace period to account for a possible delay between vaccination and recording by a healthcare professional. In conclusion, the high degree of missingness in date of onset is a challenge. We used imputation to trade systematic error for random error. We believe this was preferable to a complete-information analysis.

**Table S3. Time from vaccination to report submission for 15,353 AE terms, stratified by completeness of AE term onset date information.**

| **AE term onset date completeness** | **Time from vaccination to AE report submission, median days (IQR)** | **Number of reported AE terms** |
| --- | --- | --- |
| AE terms with complete date of onset. | 909 (385-1749) | 3882 |
| AE terms with the month and year of onset | 1183 (769-1972) | 3027 |
| AE terms with only year of onset | 1538 (979-2349) | 2844 |
| AE terms with no onset date | 1532 (928-2380) | 5600 |

Note that the number of AE terms here differs from the total AE terms in ‘Methods’ used for cluster analysis (15,949 AE terms), because for date calculations we excluded AE terms when onset date was prior to vaccination date (therefore incompatible), and reports where vaccination date was missing.
